# Megatrends in Healthcare: Review for the Swiss National Science Foundation’s National Research Programme 74 (NRP74) “Smarter Health Care”

**DOI:** 10.3389/phrs.2022.1604434

**Published:** 2022-03-22

**Authors:** Michael J. Deml, Katharina Tabea Jungo, Maud Maessen, Andrea Martani, Agne Ulyte

**Affiliations:** ^1^ Department of Sociology, Institute of Sociological Research, University of Geneva, Geneva, Switzerland; ^2^ Institute of Primary Health Care (BIHAM), University of Bern, Bern, Switzerland; ^3^ University Centre for Palliative Care, Inselspital University Hospital Bern, Bern, Switzerland; ^4^ Institute of Social and Preventive Medicine, University of Bern, Bern, Switzerland; ^5^ Institute for Biomedical Ethics (IBMB), University of Basel, Basel, Switzerland; ^6^ Epidemiology, Biostatistics and Prevention Institute (EBPI), University of Zurich, Zurich, Switzerland; ^7^ Population Heath Laboratory (#PopHealthLab), University of Fribourg, Fribourg, Switzerland

**Keywords:** healthcare, Switzerland, megatrends, national research programme 74, smarter health care

## Abstract

**Objectives:** In this paper, we present a review of some relevant megatrends in healthcare conducted as part of the Swiss National Science Foundation’s National Research Programme 74 (NRP74) “Smarter Health Care.” Our aim is to stimulate discussions about long-term tendencies underlying the current and future development of the healthcare system.

**Methods:** Our team—a multidisciplinary panel of researchers involved in the NRP74—went through an iterative process of internal consultations followed by a rapid literature review with the goal of reaching group consensus concerning the most relevant megatrends in healthcare.

**Results:** Five megatrends were identified, namely: 1) Socio-demographic shifts. 2) Broadening meaning of “health.” 3) Empowered patients and service users. 4) Digitalization in healthcare. 5) Emergence of new models of care. The main features of each megatrend are presented, drawing often on the situation in Switzerland as a paradigmatic example and adding reflections on the potential influence of the COVID-19 pandemic on them.

**Conclusion:** Considering the long-term megatrends affecting the evolution of healthcare is important—amongst other things–to understand and contextualise the relevance and implications of innovative health services research results.

## Introduction

In 2020, the Swiss National Science Foundation’s National Research Programme 74 (NRP74)^1^ “Smarter Health Care” entered its fourth year of research. Since its beginning, the NRP74 was future-oriented, in that it was tasked with three main goals [1]: 1) promote innovative health services research to tackle practical challenges related to caring for the chronically ill in Switzerland in the coming years; 2) reflect on how to improve the future health data landscape of the country; and 3) promote a novel paradigm of knowledge transfer between health research and policymaking for the development of the care system. This intrinsic attention towards the future was reinforced by the onset of the COVID-19 pandemic, which has urged even more the diverse body of researchers and clinicians within the 34 NRP74 projects to think about the evolution of healthcare, both in Switzerland and abroad. Even before the pandemic struck, the NRP74 Steering Committee had started elaborating internal reports to synthesize the research outputs of the 34 projects in order to generate future recommendations in line with the current and future healthcare trends and needs of the population. After the onset of the pandemic, the Steering Committee—as well as the broader community of NRP74 researchers—deemed it relevant, in order to deliver useful final recommendations on how to make care “Smarter,” to identify the most relevant megatrends in healthcare upon which smaller modifications in the medical system are embedded.

For these reasons, the Steering Committee asked a group of scientists from different NRP74 projects to identify the major healthcare megatrends that are necessary to consider whilst summarising evidence for research and delivering it to policymakers to determine the evolution of the healthcare system. This article provides an overview of the *megatrends* in healthcare which have been identified as a result of this effort. Our objective is to submit to a broader audience our exploration of the megatrends permeating healthcare, in order to provide discussion points and stimulate future debates on the long-term tendencies that underlie the evolution of healthcare, both at a national and international level. To do so, we describe the five megatrends identified during this process. We outline their relevance beyond the Swiss context, but we often draw on exemplary references from Switzerland. Moreover, we also consider how the pandemic interplays with the identified megatrends. Finally, we conclude by commenting on the importance of considering megatrends in discussions about the design and development of healthcare systems in the future.

## Methods

This article presents the results of an internal project conducted by scientists involved in the NRP74 in order to provide the Steering Committee with an overview of the major and long-term trends underpinning the current and future evolution of healthcare. We teamed up as a group of scientists with different disciplinary expertise, including public health, sociology, epidemiology, medicine, and bioethics. We had already been conducting research in several projects of the NRP74 and had been involved in the above-mentioned synthesis report efforts, including partaking in conferences discussing the future of healthcare service research and in dialogues with stakeholders from several institutions involved in the delivery of healthcare.

In order to identify megatrends in healthcare, we first set up a roadmap on how to perform our search. We agreed on a definition of megatrends that would guide our investigation, based on the work of the *Zukunftsinstitut*, which defines megatrends as trends or tendencies that are characterized by their long-lasting, global, and complex nature [2]. Subsequently, we searched the literature in our own fields of expertise to pinpoint trends and tendencies within healthcare that would satisfy this definition. Each author independently compiled a list of candidates to be potentially included as megatrends. We then met to present each other our findings, analyse overlaps and gaps amongst our lists, and reach agreement as to which trends would satisfy our definition of megatrend. We continued discussion until unanimous consensus was reached for the final five megatrends. Thereafter, we performed a literature review on each of the megatrends to describe their main features, relevance within and beyond the Swiss context, and the potential impact of the COVID-19 pandemic.

According to the taxonomy of reviews proposed by Grant and Booth [3], our process corresponds to a mapping review, which is characterised by the aim of sketching knowledge around a certain topic (in our case, megatrends in healthcare) to offer “policymakers, practitioners and researchers an explicit and transparent means of identifying narrower policy and practice-relevant review questions” (p. 97). During the identifications of the main features of the selected megatrends from the literature, we had repeated internal consultations among our research team to ensure an accurate depiction of each megatrend. This process was conducted between April and June 2021, after which the main findings of our search were presented to the NRP74 Steering committee. Thereafter, we further refined our findings and assembled it in the current article.

In the following results section, we present the megatrends identified through this process, by reviewing their main features and their significance for healthcare. We often reference Switzerland as a paradigmatic case where such megatrends are unfolding and we also reflect on the influence that the pandemic might have on them.

## Results

**FIGURE 1:**
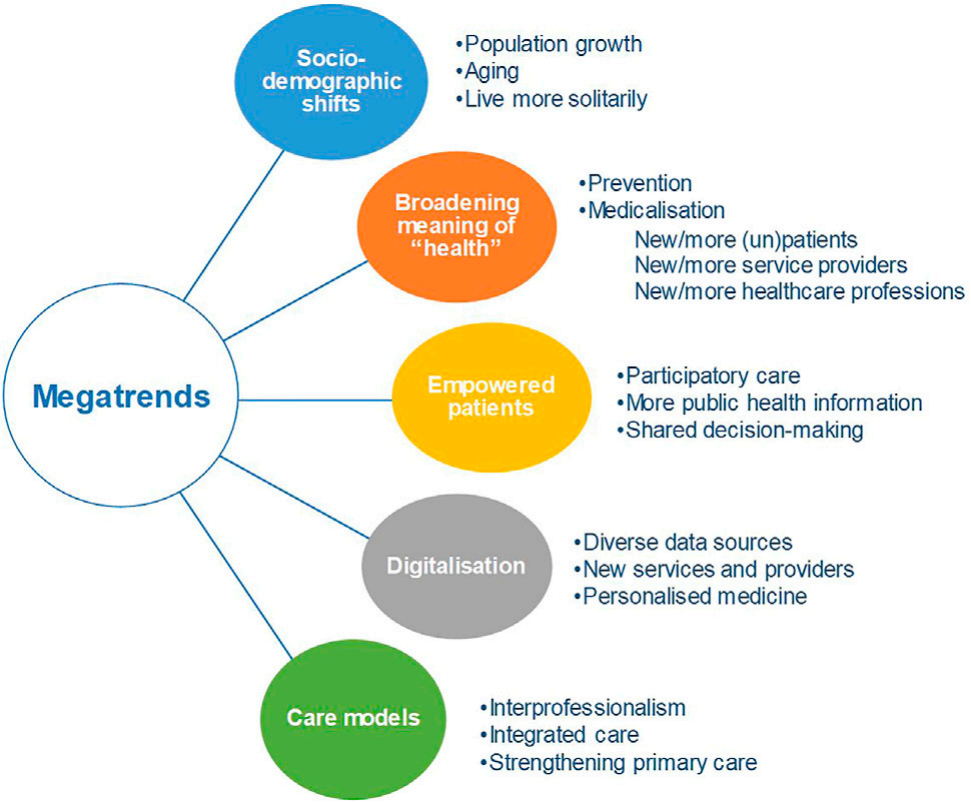
Overview of megatrends that will likely be relevant in the coming years for Swiss healthcare (Switzerland. 2021).

This study identified the following megatrends in healthcare, which are here listed succinctly and presented graphically (Figure 1) to then be more extensively described below:

1) socio-demographic shifts, such as an aging population, migration, employment, and urbanisation of certain regions, and potential implications for healthcare.
2) broadening definitions of “health” and “patients,” which have repercussions regarding the medicalisation of society and opportunities for new markets in healthcare.
3) the issues we face when a context of an *infodemic* is coupled with previous health promotion and public health efforts having encouraged patients to be informed, empowered and to participate in shared decision-making with clinicians.
4) digitalisation in healthcare and the potential issues moving forward, such as the use of these data, privacy, and interoperability.
(5) changes in care and care models, such as the increased use of communication tools, innovative care models, interprofessionalism, and strengthening primary care settings.

### Socio-Demographic Shifts

The first megatrend that we identified concerns socio-demographic shifts. Long term socio-demographic changes are common and universal and have a direct impact on the health care needs of a population. Here we refer to Switzerland as a paradigmatic case, as it resembles shifts in other countries.

The population in Switzerland is experiencing multiple demographic transitions, including population growth, urbanisation and household composition. According to the Swiss Federal Statistical Office, Switzerland’s number of permanent residents will rise by 22%, from 8.7 million people in 2020, to 10.4 million^2^ in 2050 [4]. Urban areas, such as the Cantons of Geneva and Aargau, will show the strongest population growth. In more rural areas, such as the Cantons of Ticino and Graubünden, a 5% decrease is expected by 2050. In 2050, it is also projected that 23% more people will live alone in their homes compared to 2020. In total, this will be almost half of the population (4.7 million) [4].

The driving force of the increasing number of Swiss residents is migration. In general, migration in Switzerland increases the number of young adults. However, the proportion of permanent residents aged 20–64 in the total population is expected to decrease from 62% in 2018 to 55% in 2050. This is mainly the result of population aging. The number of permanent residents aged 65 and older is expected to increase from 1.6 million in 2018 (18%) to 2.7 million in 2050 (26%). Not included in the migration numbers are the people who work in Switzerland but have their permanent residence in a neighbouring country. This number is expected to grow from 337,000 in 2020 to 526,000 in 2050 [4].

The above-described projections for socio-demographic shifts imply that the population potentially eligible for employment will decrease, which will likely increase the financial pressure on the total Swiss social security system. Old age insurance in particular will face financial challenges, as the number of potentially retired persons (aged 65 and older) per 100 people aged 20–64 is expected to grow from 36 in 2020 to 53 by 2050 [5].

Permanent residents of Switzerland aged 80 years or older will increase by 50% between 2020 and 2050 [4]. The growing old-age population and the longer life expectancy will heighten the need for healthcare services, especially for complex multimorbid care of the chronically ill and for palliative care. Moreover, the need for home care services and long-term care can be expected to increase. The pressure on these services is further intensified as a result of the decreasing availability of family caregivers due to the rising number of patients living alone, family not living close, and potential family caregivers being unable to combine informal caregiving and employment [6].

In addition, socio-demographic shifts also directly challenge the healthcare provision [7]. For example, care institutions are testing care models for care provision that better accommodates the diversity of cultural and language preferences in the growing multicultural society [8]. It is reasonable to expect particular challenges in culturally complex issues related to deteriorating health, death, and grief. The increasing care utilisation and accompanying cost requires innovative healthcare system solutions, such as complex care management [9, 10], and managed care [11].

The effects of the COVID-19 pandemic on such demographic shifts are currently unknown. The pandemic could have an effect on the described aging and migration rates. However, the currently^3^ reported annual COVID-19 related deaths (11,200 in Switzerland) do not indicate a substantial nor long-term influence [12].

### The Broadening Meaning of “Health”

A further megatrend influencing the domain of healthcare both in Switzerland and abroad concerns the changing meaning and the widening scope of what counts as “health.” Indeed, advances in the fields of genetics and the development of preventive approaches to medicine such as P4 medicine (predictive, personalised, preventive, participatory) [13] are channelling the focus of the healthcare sector to lifestyle and to the way health is pre-emptively promoted. This differs from previous, traditional approaches that instead focused on how illnesses used to be treated only when they would manifest themselves. Such a shift is embodied by new definitions of health, which insist on the existence of a biologically given and a personally acquired component, and argue for the focus of healthcare to move from the former to the latter [14].

In this sense, the traditional distinction between patients and healthy individuals is becoming more and more blurred. This is due to the tendency of expanding the definitions of diseases, which allow to identify individuals as “new patients” more easily, although it remains unclear if early detection ultimately benefits them [15]. Some have even argued that healthcare is increasingly concerning not only patients, but also “unpatients.” This term refers to individuals who are “neither patients in the usual sense of being under treatment, nor nonpatients, in the sense of being free of a medically relevant condition,” because they have risk factors or “genetic predispositions [indicating that] some condition may come to them but [they will not know] precisely if, when and how” (p. 623) [16]. This influences also the vision many stakeholders have for the future of healthcare, which foresees that “health is done at home, hospitals are only for repairs” (our translation) [17].

The broadening of the scope of healthcare is also generating new market spaces where companies have started to invest and thrive. In addition to, and sometimes in alliance with, traditional big-pharma industry, many big tech companies now contribute to the creation of new tools for treatment, prevention, and/or for the maintenance of good health [18]. The involvement of tech-companies^4^ in the public health response to the COVID-19 pandemic both in Switzerland and abroad (which has been described as a “Googlization” of the pandemic response [20]) is a telling example of the growing interest of such industries in the field of health.

A further sign of the involvement of new companies into healthcare is the growing number of digital therapeutics developed for the management of chronic diseases [21]. *Digital therapeutics* refers to the “new treatment modality in which digital systems (e.g., smartphone apps) are used as regulatory body-approved, prescribed therapeutic interventions to treat medical conditions” [22]. For example, smartwatch apps have been tested as tools to help diagnose atrial fibrillation [23]. Such tools often represent new sources of real-time health data streams, which adds to the challenges raised by the megatrend of digitalisation.

The development of digital therapeutics and the increased involvement of non-medical companies in the field of healthcare have also contributed to the phenomenon of medicalisation in new parts of life. Medicalisation refers to the process through which “each person’s whole dynamic life process is defined in biomedical, technoscientific terms as controllable and underlain a regime of control in terms of monitoring, quantification, prediction, risk profiling, early diagnosis, therapy, prevention and optimization that is all-encompassing.” [24] In practice, this means that the scope of ‘what healthcare is about’ has further expanded. This raises questions about whether medicalizing further parts of lives is a purely medical development or a value-laden process, as illustrated by the debate around prenatal genetic testing and screening [25].

Such trends create the possibility for new professions that provide services that are thought of as part of what healthcare systems should offer. For example, with the increasing availability of genetic testing, genetic counsellors (healthcare professionals helping patients presenting a condition with a genetic component) are now present in many countries [26], and their occupation is becoming further professionalised [27]. Similarly, there have been calls to develop health information counsellors [28] (professional figures to help individuals navigate through the increasing amount of digital data that are collected about them with a bearing on their health). At the same time, the medicalisation of old-age has led to the establishment in countries like Switzerland of advance care planners (experts who help take the medically-relevant decisions about the last part of life) [29]. Lastly, the expanding scope of health is prompting not only the emergence of new professions in the care system, but also the increasing involvement of non-medical disciplines in research about healthcare, such as behavioural science, as it has recently been discussed with reference to Switzerland [30].

### Informed, Empowered Patients, Shared Decision-Making and the “Infodemic”

Intersecting social trends, public health efforts, and normative expectations have collectively constructed the figure of autonomous, empowered, and self-informing patients. This has been further catalysed by the coronavirus pandemic. Indeed, patients are increasingly exhibiting, and expected to exhibit, behaviours of what popular and research discourse consider to be the characteristics of “good patients.” Several studies have considered clinicians’ expectations for positive behaviours of “good patients,” including: knowledgeable and informed patients, patients who ask questions, patients who comply/adhere to clinicians’ recommendations [31], those who are engaged and interactive, those who do not have an illness caused by “bad habits,” and those who are motivated to improve their health status [32].

This trend of the involved, informed, and autonomous patient can be traced back to when the concept of *health promotion* arrived to the healthcare agenda. For example, in the World Health Organization’s 1986 *Ottawa Charter for Health Promotion*, an emphasis was placed on encouraging individuals to take health into their own hands: “People cannot achieve their fullest health potential unless they are able to take control of those things which determine their health” (p. 1) [33]. The underlying idea is that patients are, in this line of thinking, expected to exercise greater levels of agency, or “individual autonomous action” (p. 163) [34] in making health-related decisions.

The trend of increasing expectations for patients to be more empowered by informing themselves and being active participants in their healthcare decisions has far-reaching implications for healthcare. In effect, the ongoing pandemic has brought to the fore tensions involved when experts and patients alike learn important medical information virtually simultaneously. The constant influx of information—and ease with which people can access, engage with, and misinterpret new information—has prompted the World Health Organization to refer to these issues as an *infodemic*, which is defined as “too much information including false or misleading information in digital and physical environments during a disease outbreak” [35]. Concerns about misinformation in the context of the pandemic also prompted the Swiss National Science Foundation Task Force to issue a policy brief entitled “Response to corona denial” [36].

In the context of a health infodemic, we face issues related to people’s understanding of the abundance of available information. In the fields of public health, health promotion, and health intervention, we have also seen well-intended efforts to improve “health literacy”^5^ as a desired outcome, which remains in line with the idea that patients should be empowered to take ownership for their own health. However, the noble goal of increasing people’s health literacy as a mediator of improving collective health and well-being through individual actions can be hindered by issues such as the rising importance of information seeking-behaviours of individuals who look for information online, with the advent of Dr Google [38], information overload [39], misinformation [40], health forums online providing advice running contra to biomedical recommendations [41], and health social movements which may contest conventional biomedical consensus [42].

We have also seen movements in recent years encouraging clinicians to engage in “shared decision-making” during consultations. Shared decision-making has been defined as “as a decision-making process jointly shared by patients and their health care providers” (p. 526) [43]. The encouragement of shared decision-making, combined with autonomous, informed patients, can sometimes elicit dilemmas for clinicians during consultations when patients disagree with the desired clinical outcome encouraged by clinicians [44].

The trend of the informed, knowledgeable, and autonomous patient is likely to shape future dynamics in patient-provider relationships. The pandemic has demonstrated how patients’ roles are changing when patients and healthcare professionals alike have an abundance of information available to them.

### Digitalisation and Data in Healthcare

Digitalisation of healthcare data—or the recording of clinical information electronically and using digital technologies to manage it - and the recognition of digital data as an asset have numerous implications for patients, healthcare providers, and industry stakeholders. Two major differences between digital and paper-recorded healthcare data are related to their potential accessibility and possibility to directly analyse patterns automatically.

Digitalisation has been a consistent process over the past few decades, although in the healthcare sector it lags behind compared to other industries and public services due to legal constraints, lack of integration of different data infrastructures, incentives, and perceived benefit by providers [45–47]. In Switzerland, 34% of general practitioners were still using exclusively paper health records in 2015 [48]. Expectations for the possibilities made available through digitalisation stood in stark contrast to the reality of the situation during the COVID-19 pandemic, with case reporting occurring via fax [49] and mistakes in the documentation of casualties [50]. Data in healthcare are diverse: some data points, such as diagnosis and procedure codes, are quick to become digital if they are to be collected for billing purposes. Others, such as consultation notes, take more time to be digitalised, especially if incentives and direct benefits for providers are lacking.

Digital health data facilitate the analysis of its patterns at the patient and population levels. At the patient level, this enables the development of personalised medicine, by integrating all data points of a single patient for diagnosis and prediction purposes [51]. Digital health data could allow earlier detection of health conditions, finer prediction of success of a specific treatment, and more direct monitoring of patient-logged or automatically collected health parameters. Patients are increasingly expecting these data to be integrated and used in their healthcare [52]. At the population level, researchers can use digital health data to identify real-world treatment effects, including rare adverse effects. Such evidence can be generated in almost real time. The speed of generating evidence has proven to be especially important during the COVID-19 pandemic, although it has also raised concerns about the rigour of research and trustworthiness of the data sources [53, 54]. Although versatile for descriptive and predictive tasks, observational data require more complex analysis methods in order to, under certain conditions, lead to valid causal conclusions [55]. Insights from digital healthcare data are also limited if the varied sources cannot be linked and integrated, that is, if they are not interoperable [56].

Different solutions have emerged for efficient, privacy-protecting research with healthcare data, such as national linking infrastructure [57] and decentralised analysis approach [58]. Big healthcare data—consisting of numerous observation points and recorded features (dimensions)—have led to high interest in machine learning applications. The latter are applied most prominently for the analysis of non-structured data, such as free-text consultation notes and radiological images. Despite the promise, the deployment of artificial intelligence-based tools has been slower than expected due to methodological and legal challenges, lack of clinical evaluation and issues with implementation [59]. Their implementation will likely require novel approval and oversight procedures, different from those used for current healthcare hardware and even software [60].

The digitalisation of healthcare data is transforming how services are provided. Decision-support systems can be integrated into electronic healthcare systems, with a potential to reduce mistakes and increase guideline adherence [61]. Many routine tasks in healthcare can be automated, from primary [62] to highly specialised care, such as supervising ventilated patients at intensive care units. Direct-to-patients telehealth, teleconsultations between providers, and telemonitoring have further gained popularity during the pandemic [63]. In a data- and algorithm-saturated healthcare environment, providers will need to acquire new skills. On the other hand, relieving them from tasks that can be automated creates the potential for more value-based care and more time in direct contact with patients.

Finally, digitalisation of healthcare data has invited a new kind of health service provider to the table: data-driven tech companies. Companies, whose business models rely on analysing the patterns of massive amounts of personal data generated by their users, are keen to enter the healthcare market, although they are not always able to reliably demonstrate their added value to patients and public [64, 65]. Involvement of commercial companies with a track record of data misuse raises privacy concerns [66, 67]. Concerns are particularly high when sensitive health data are used by non-healthcare providers and regulators (e.g., vaccination passports for travelling and accessing non-health services), and flaws in data systems are discovered [68].

### Changes in Care and Care Models

In past decades, various changes in how healthcare is organised and provided have started to take place. Some of the changes described below have become more important during the COVID-19 pandemic.

First, several changes related to the provision of care are linked to the above-described megatrend of digitalisation. As a result of the development of innovative digital communication tools, new ways of patient-provider interactions have become possible. For instance, telemedicine and telemonitoring have become more accessible and more commonly used tools in the healthcare sector [69]. Particularly during the COVID-19 pandemic, their use was accelerated, and it is imaginable that both telemedicine and telemonitoring will remain commonly used in post-COVID-19 times [63]. Moreover, the availability of new eHealth tools, such as mobile apps or internet-based programs to be used for patients, provide new possibilities for patients to self-manage their conditions, symptoms and adherence to treatment plans [70].

Second, there has been a shift towards innovative care models that transform the ways in which healthcare is currently delivered. Despite a slower start in Switzerland than elsewhere, the number of integrated care initiatives has increased [71]. Despite a lack of consensus on its definition, integrated care is often used as a synonym of “coordinated care” [72]) or care integration, which can be defined as a “coherent set of methods and models on the funding, administrative, organisational, service delivery and clinical levels designed to create connectivity, alignment and collaboration within and between the cure and care sectors” [73]. The goal of integrated care models is to combat fragmentation of care delivery and associated additional costs. Additionally, in the context of population aging and related demographic shifts, long-term care models will become more important [74]. In many European countries, there is a trend towards home-based care, which allows older adults to live independently as long as possible [75].

Third, to overcome provider silos that exist in the healthcare system and to reduce inefficiencies, there is a trend towards interprofessionalism [76], which consists in the collaboration between different professions who have different knowledge, skills and abilities. As a consequence, working in interprofessional, collaborative care teams, which also include patients and informal caregivers, rather than parallel work structures, is becoming increasingly popular [77]. Successfully integrating interprofessional collaboration in care requires overcoming several challenges (e.g., communication between different health professions, or integrating ways of how to work interprofessionally in the education of different health professions) [78].

Fourth, despite the majority of patient-provider contacts taking place in the primary care setting, the latter usually receives less attention than the secondary and tertiary care settings (for instance in terms of funding) [79]. Indeed, increased investments in primary care lead to positive population health outcomes, reduced secondary care usage, as well as reduced overall health costs [80–83]. Through the First International Conference on Primary Healthcare and the signing of the Astana Declaration in 2018, there was a joint global effort to strengthen primary care [84]. The COVID-19 pandemic showed that strong primary care is crucial for population health (e.g., testing in the community, treating community members with COVID-19 or long COVID, vaccination) [85]. Strengthening primary care is thus an important trend in future healthcare systems.

Fifth, in the context of increasing rates of chronic disease and ageing populations, rehabilitation becomes increasingly important. As stated in the Rehabilitation 2030 Initiative of the World Health Organization (WHO), rehabilitation should be made available for everyone throughout the life-course and it should be integrated in all levels of healthcare [86]. In light of the large number of individuals who suffer from long-term physical and psychological symptoms after an infection with the coronavirus [87], rehabilitation efforts are crucial. Long Covid rehabilitation services have become available at numerous rehabilitation facilities in Switzerland [88].

### Limitations

Our review has some evident limitations. First, it is based on work which we conducted as part of the engagement of the NRP74 to improve Swiss healthcare, hence the megatrends identified are certainly skewed by the perspective of a western and high-income country. For this reason, we acknowledge that the megatrends herein identified—despite bearing relevance beyond the Swiss context—are articulated in a way that will probably resonate with more familiarity (but not exclusively) with a European readership. Moreover, the selection of the megatrends and the review of the literature were done in a non-systematic way. Nevertheless, this corresponds to the features of a mapping review, whose main aim is, quite literally, that of “charting the territory” around a general topic. Furthermore, by elucidating the process by which we approached the identification of megatrends, we provided transparency as to how we approached this review.

## Conclusions

Around the world, many research projects are continuously created with the aim to influence the future of healthcare in their respective context, just like the NRP74 has done in Switzerland in the last few years. However, when trying to steer the evolution of healthcare, it is important that single policy modifications do not neglect the long-term trends in which they are embedded. In this article, we presented some of the most relevant megatrends in healthcare to foster awareness around the most far-reaching tendencies underlying developments in this sector. Far from being exhaustive, our list of megatrends is aimed at stimulating further debate on the historical evolution of how healthcare is conceived. The changing nature and function fulfilled by healthcare are also bound to generate new ethical questions, both at a societal level and at the bedside. For example, the increased exchange of patient data between healthcare professionals has called into question our understanding of medical confidentiality; or else, the rhetoric of patient empowerment has fortunately changed the traditional paternalistic orientation of medicine, but it has also shown some limits when translated in the public health context (e.g., in the context of lockdown measures against an epidemic, when the choices of individuals have a direct bearing on the welfare of others). In this respect, we believe that reflecting on the megatrends and their historical antecedents is particularly important at a time where the current COVID-19 pandemic has the potential to influence pre-existing trends and to change healthcare trajectories. To try and ensure that we are active drivers—rather than passive subjects—of the evolution of healthcare, it is crucial to keep an eye on the most significant trends that are shaping it. It is also equally important to promote health services research (e.g., effectiveness studies, implementation studies, health economic analyses), which is a powerful instrument to understand the transformations taking place in the medical sector. The field of health services research is relatively young and underdeveloped in Switzerland, in comparison with other countries [89]. In this respect, we trust that more attention towards the megatrends in healthcare—and to the influence that events like the COVID-19 pandemic can have thereon—can be a driver to learn for this kind of research, so that the newest developments in healthcare can be reliably understood and evaluated.

## Footnotes

1
http://www.nfp74.ch/en, accessed 03.12.2021.

2 Permanent residents include all Swiss nationals with their main residence in Switzerland, but also foreign nationals with a residence or settlement permit for at least 12 months, foreign nationals with a short-term residence permit for a cumulative duration of stay of at least 12 months, and persons in the asylum process with a total duration of stay of at least 12 months.

3 As of December 2021, as per the report referenced hereafter.

4 The involvement of Google and Apple with respect to contact tracing apps is described, for example, here [19].

5 For terminological considerations and possible definitions, see [37].
